# Exploring Reproductive Health Impact of COVID 19 pandemic: In Depth Interviews with key stakeholders in Pakistan

**DOI:** 10.12669/pjms.37.4.3877

**Published:** 2021

**Authors:** Nighat Shah, Mehjabeen Musharraf, Farah Khan, Nusrat Shah

**Affiliations:** 1 Dr. Nighat Shah Assistant Professor, Jinnah Sindh Medical University, Karachi, Pakistan; 2 Mehjabeen Musharraf, Senior lecturer, Jinnah Sindh Medical University, Karachi, Pakistan; 3 Dr. Farah Khan Senior Instructor, Jinnah Sindh Medical University, Karachi, Pakistan; 4 Dr. Nusrat Shah Professor OBGYN – DUHS, Civil Hospital, Karachi, Pakistan

**Keywords:** COVID 19, Pandemic, RH, FP, Lock down, Qualitative study

## Abstract

**Objective:**

To explore the effects of COVID 19 on reproductive and child health services and gender relations.

**Methods:**

This is a Qualitative Exploratory Research. Due to lockdown, setting was online interviews on Zoom. Sampling was purposive. Five in-depth interviews were conducted in June 2020 followed by compilation of results and manuscript writing in July and August 2020.

**Results:**

Maternal Neonatal morbidity and mortality will rise as part of collateral damage of C19. As all routine services of maternity care, family planning, post abortion care and vaccination were also in lockdown. Baby boom, unwanted pregnancies, unsafe abortions and violence against women will be the secondary consequences of C19.

**Conclusion:**

Some critical services should never stop which include maternal and neonatal essential services. MNCH service continuity has to be maintained to optimize maternal neonatal health, prevent unwanted pregnancy and abortion. With appropriate standard operating procedures, and protective equipments, health facilities need to open. LHWs and community mobilisers with PPEs should continue services.

## INTRODUCTION

Corona virus has adversely affected all specialties including reproductive health (RH) services especially in low income countries.^1^ Lockdown on services like reproductive health will result in more unwanted pregnancies, unsafe abortions and maternal mortality. According to UNFPA (28th April 2020), more than 47 million women could lose access to contraception, leading to 7 million unintended pregnancies in the coming months.^2^

More than half the world is under lockdown, contraceptive commodities are deficient including condoms.^3^ In many countries FP and abortion care are not essential services, hence care providers are unable to provide required lifesaving services.^4^,^5^ Moreover, neonates and children will suffer as 4.5 million children are missing vaccination in South Asia. Many NGOS like green star social marketing and Marie stopes are closed in countries like Pakistan, India and Nepal.^4^

In Pakistan, with a weak healthcare infrastructure, government is trying to cope with the extra burden of COVID19.^6^ Due to this, the healthcare professionals are stressed out, laboratories, emergency rooms and ICUs are not meeting the needs of infected patients.^6^ Karachi is on the lowest rate of immunization as compared to other mega cities of Pakistan and during lockdown, on average, 2734 children missed the immunization in Karachi.^7^ We don’t have local studies available regarding the impact of lockdown and this pandemic on RH services; therefore, we undertook to grasp the stakes and consequences of C19 on Maternal and neonatal health, if government policies during the pandemic do not ensure that sexual and reproductive health services continue. Thus, the study aims to explore the effects of COVID 19 on reproductive and child health services and gender relations.

## METHODS

After Ethical approval from JSMU (JSMU/ IRB/ 2020/ -333) on May 29^th^, 2020, experts of Public health were purposefully selected and briefed about the purpose of study on phone prior to scheduling a zoom call. There were total of five participants; two females and three males, mean years of experience is 25 years and they all have post graduation qualification in Medicine. Designations of participants are CEO, Chief of Party, Senior Advocacy Consultant, founder of a hospital and Technical Advisor. COREQ guidelines have been followed for this research. Interview guide was used with few open-ended questions to prompt the discussion followed by probing. The questions were: How has this Pandemic affected your work? (gaps in work, backlog). How has COVID – 19 affected women? How COVID- 19 has affected the women’s reproductive and sexual health in general?

Setting was on-line interviews from the important RH stakeholders of Karachi. Karachi is a densely populated metropolitan city of various ethnicities and religions with a population around 16.1 million. Health system of Karachi mostly depends on out of the pocket expenditure with few public sector tertiary care hospitals which do not cover the health care needs of this big city. Data collection started in June and compilation of results and manuscript writing in July and August 2020. The interviews were recorded, verbatim transcription was done and notes of co-authors were also incorporated. The duration of IDIs was 30 – 45 minutes. PI is a gynecologist and teaches RH to MSPH. She conceived the idea, identified the participants, conducted in-depth interviews and performed analysis. She was responsible for the overall accuracy of the work. Second author has expertise in Qualitative Research and did analysis and result compilation. Third author has special interest in family planning and Reproductive medicine. She wrote memos and discussion. Fourth author is a Professor, educationist and a researcher. She designed the interview guide and helped in manuscript writing. Authors are trained in conducting qualitative research and analyzing the data from APPNA Institute of Public Health, Jinnah Sindh Medical University. PI has attempted to bracket out her predetermined notions to achieve the conformability which is one of the four constructs of trustworthiness given by Guba.^8^ According to her, there is an acute neglect of maternal and child health due to resource reallocation to COVID-19 pandemic and the FP services closure has resulted in more unintended pregnancies and abortions.

To ensure the transferability, setting has been briefly described so that readers can relate the findings with the similar settings. Analysis of the data started side-by-side with data collection and credibility was achieved by investigator’s triangulation that is two authors did the analysis. After analyzing the first two transcripts separately, the researchers discussed open codes and the categorization of codes. The audit trail of the same is shown in Table-I. Later, all transcripts were deductively analyzed separately and then with consensus the sorted data in each theme were finalized.

**Table-I:** Audit trail of theme generation.

Categorization of codes	Sub themes	Themes
Routine Maternal and child health centers closed Antenatal Care minimal Labor and delivery affected Many private maternity centers closed FP not an essential service, not available during lockdown Family planning commodities not available Home deliveries increase All budget and efforts in COVID MNCH affected (service delivery, supply chain) LHWs are no more doing their work. Promotion of traditional birth attendant	Inaccessibility/lack of supplies Unavailability of services in hospitals/centers Rise of TBAs Diversion of RH Budget	**How COVID-19 affected MNCH services**
LHWs should continue with PPEs Hospitals to follow SOPs Safety of health care providers by personal protective equipments Availability of emergency contraceptive pills Tele-medicine and public awareness campaigns		Adoption of New Normal
Women stay at homes so less chances of exposure Mortality and morbidity in women would be higher as they are anemic immunity, nutritional status is poor Gender-based violence at homes Men are homes 24 hours Perpetrators are husband and relatives Frustration from men	Social aspects Biological aspects	COVID 19: A Gender lens
Government activities Formulation of guidelines Continuation of FP and RH services FPRH group		Positive Developments

## RESULTS

### Theme I:

How COVID-19 affected MNCH services

### 1) Inaccessibility/lack of supplies

Regarding a shortage of essential supplies like family planning commodities and MVA kits, two contrasting opinions came. One opinion is that the stock is short in some centres so there is a need for shuffling. According to the second opinion, we have completely run out of family planning commodities.

Supply of FP commodities are badly affected and 50 % of facilities do not have the supplies. Imagine a woman who is on injections and if she doesn’t get her next injection; she obviously will get pregnant. (NA, Chief of Party, JSI)

Since the chemist is closed ECPs are not available, so we attempted to distribute the commodities (condoms, IUCD, misoprostol). We were blocked by security officers all over Pakistan and our mobile units were sent back. (AR, CEO, Green star Social Marketing)

### 2. Unavailability of services in hospitals/centers

***“WHO put Family Planning in the seven essential SRHR services, but when it comes to crisis it isn’t listed as essential service. FP clinics are therefore closed in this Pandemic (YS, Senior Advocacy Consultant to Gates Foundation)***

Whole situation of Covid will add to the morbidity and mortality of women due to the unavailability of services. The lady health workers who used to go door to door for maternal and childcare are also restricted.

***Hospitals in Karachi are now receiving more pregnancy-related medical and surgical emergencies than before from all over Sindh due to mobility issues and reaching late to the health facility. (SS, Founder of Koohi Goth Hospital)***

### 3. Rise of TBAs

Participants shared their strong reservation that the current situation will promote the traditional birth attendant.

***One who has to come will come to this world, so a woman would require these services. So what are we promoting? that they go back to the same traditional birth attendants, or if there are complications where can they go? (NA, Chief of Party, JSI)***

***TBAs have become very active now because women are unable to go to facilities, so how do we mitigate that risk? And we do know that TBA s are risky with no infection prevention practices. (YS, Senior Advocacy Consultant to Gates Foundation)***

### 4. Diversion of RH Budget

Current situation has caused the donors to repurpose the budget to meet Covid requirements due to which other activities get compromised.

***The ongoing battle is to make donors understand that we cannot allocate the whole budget on covid as we have such low indicators for maternal and child care in Pakistan. After Covid, there will be a baby boom. (YS, Senior Advocacy Consultant to Gates Foundation)***

### Theme II: Adoption of New Normal

Participants stressed upon the role of the department of health to make sure that the reproductive services are not denied.

LHW program for home health should continue with PPEs. They can guide for care of Covid 19 positive women, regarding breastfeeding and necessary required education.

***Guidelines made by local public health leaders for “RH in covid era” should be adopted and handed over to healthcare providers. We have to take extraordinary measures instead of just shutting down everything. (NA, Chief of Party, JSI)***

Availability of emergency contraceptive pills should be ensured. This will help avoid many unwanted pregnancies and abortions. Tele-medicine, public awareness campaigns should be used for health promotion.

### Theme III: COVID 19: A Gender lens

### 1. Social aspects

As men are not going out for work and are thus frustrated this increases the violence on women.

***Women generally suffer more and battered by men who are not well educated. But I have seen that in educated families also so it is not surprising that gender based violence will increase (NA, Chief of Party, JSI)***

***Pakistan already is among the top countries facing gender-based violence. Hence sexual violence like marital rape and women becoming pregnant unintentionally will increase. (YS, Senior Advocacy Consultant to Gates Foundation)***

Providers are not trained on recognizing and referring the sensitive cases of domestic violence. However, such cases must be dealt with care because such women are disturbed, depressed and anxious.

### 2. Biological aspects

Participants said that considering the social set-up of Pakistan majority of the women stay at homes and are less exposed. According to one participant, in comparison to men, women would be affected more because they are anemic and the immunity and nutritional status is poor. Other narrative is that X chromosome is protective against C19, and estrogen and progesterone enhance the immunity, on the contrary in men testosterone does not contribute in strengthening the immune system.

### Theme IV: Positive developments

Government of Sindh has developed guidelines on Family Planning and Reproductive health during covid-19 with the help of development partners.

Guidelines have been issued which describe continuation of FP services. All the reproductive Health Services centers and family welfare centers will remain open in the vicinity of hospitals. RH services have decreased, but we don’t want to be complacent. We want to ensure RH services, therefore, we have developed a Covid response by making Family planning Reproductive health group of all the partners for reviewing the situation in Sindh. (TL, Technical advisor, PWD)

***(For maintaining the confidentiality, initials are given to the participants)***

## DISCUSSION

Study highlighted that the routine RH care is compromised and this is also shared by other neighboring countries like India.^9^ This will result in more unwanted pregnancies, unsafe abortions and maternal mortality as seen in other low resource countries.^10^ Experts expressed concerns regarding the next pandemic, “Baby Boom” which would be disastrous for overpopulated country like Pakistan. Similar concerns were addressed in research by Church K et al.^11^ According to UNFPA, the emergency response to COVID-19 also means that resources for sexual and reproductive health services are diverted to deal with the outbreak, contributing to a rise in maternal and newborn mortality.^12^ FP was considered the most important task assigned which if neglected will bring long term implications: Call for action has been given in other studies as well.^13^

Discussion in this research brought forth the issue of RH facilities being closed, hence leading to unwanted pregnancies which will result in complications consequently adding to maternal and neonatal mortality.^12^

Rise of unskilled birth attendants is another side effect of this pandemic according to the study’s public health leaders. As shown in many studies these practices are associated with preventable maternal and neonatal mortality and morbidity.^12^,^14^ The Ebola epidemic study highlighted maternal, neonatal deaths as indirect damage of epidemic.^15^

Whenever there is a national health catastrophe the interests of public at large supersede RH concerns. Even the advanced world is having difficulty in balancing this need.^16^ Our study experts also raised alarm that budget allocations for RH will now be diverted towards health crisis. This will result in severe deficiencies in commodities and RH work force.

As the pandemic will stay for some time, new norms have to come in. The essential services should continue. In this regard, the participants stressed upon the role of the government to ensure care of pregnant women with the incorporation of specific SOPs. A critical component of post abortion care is post abortion family planning^17^ which is sluggish due to Pandemic crises, as mentioned by study participants. Pakistan already is burdened with high unsafe abortion rate of 2.2 million.^18^

Pakistan and India already have a huge unmet need for family planning. In India 30 million women (Census 2011 data base, the unmet need data obtained from NFHS-4, 2015-16) wanting to use contraception but unable to do so. The outbreak will further deteriorate the situation.^19^ Economic break down, loss of jobs and frustration will lead to domestic violence as well globally.^19^,^20^ As far as gender perspective is concerned, males especially with known comorbidities have higher preponderance of disease and complications.^21^ The mortality of COVID is 2.4 times more in males.^22^ However, in pregnant women with altered immunity and anatomy more complications and death can occur.^23^

Study experts stressed on putting RH services as essential services**.** They gave a roadmap with guidelines for safety of mothers and neonates and continuity of care for women who are pregnant. They stressed specific SOPs to be followed for which hospital systems should be strengthened. Standard recommendations are given by WHO in this regard which needs to be implemented.^24^,^25^ Participants also said that the continuity of reproductive services should be ensured through LHWs.

After having a detailed discussion on the said topic, the technical advisor of costed implementation program, Govt of Sindh, Mr. Talib Lashari has put forward two-point agenda to the health ministry, which Government of Sindh has approved. This paper hence has following policy implications.

1. Declaration of RH services including family planning as essential
2. Allowing the community midwives to continue work

Qualitative research is meant to get the in-depth information which should preferably be done by having face to face conversations so that the greater details of the topic can be captured. Data collection of this study was done on-line and is one of the limitations. In addition, some of the contacted participants refused due to the pandemic crisis which might have compromised the descriptions of some aspects of the phenomenon.

## CONCLUSION

Lessons from our research will demonstrate the detrimental influences that can result from a health emergency. Our country need to make decisions to balance the demands of responding directly to COVID-19, while simultaneously engaging in strategic planning and coordinated action to maintain essential health service delivery, mitigating the risk of health system collapse. Therefore, the key leaders should look at making the RH services essential.

### Authors’ Contribution:

**Dr. Nighat Shah:** Conceived the idea, identified the participants, conducted in-depth interviews and performed analysis. She was responsible for the overall accuracy of the work.

**Mehjabeen Musharraf:** Did analysis and result compilation.

**Dr. Farah Khan: W**rote memos and discussion.

**Dr. Nusrat Shah:** Designed the interview guide and helped in manuscript writing.
